# Research on collaborative development mechanism of multiple stakeholders in medical institutions based on rough set

**DOI:** 10.1186/s12889-024-17813-6

**Published:** 2024-02-06

**Authors:** Mao-min Jiang, Yang Kong

**Affiliations:** 1 School of Health Management, Binzhou Medical University, Yantai, 264003 China; 2 School of Public Affairs, Xiamen University, Xiamen, 361005 China

**Keywords:** Medical institution, Patients, Doctors, Conflict analysis, Rough set, Collaborative development

## Abstract

Reducing doctor-patient conflict is an important part of coordinating doctor-patient disputes and easing doctor-patient relationship, which is conducive to building a harmonious medical environment and promoting the healthy development of medical undertakings. This paper constructs a multi-decision-maker mixed conflict model based on rough set theory, puts forward the matrix operation expression of the conflict degree theory in the Pawlak model, and gives a more objective and scientific evaluation function. Combined with hot issues of doctor-patient conflict, the proposed multi-decision-maker mixed conflict model is applied to doctor-patient conflict, examines the doctor-patient relationship in the medical institution system from multiple internal perspectives, and calculates feasible solutions in the conflict system. The results show that high medical quality, high standardize medication, high institutional efficiency, high staff efficiency, high hospital benefits, high hospital revenue, medium employee development, medium equipment development, or high medical quality, high standardize medication, high institutional efficiency, medium staff efficiency, medium hospital benefits, high hospital revenue, high employee development, and high equipment development are important conditions for building a harmonious medical environment and reducing doctor-patient conflicts.

## Introduction

In recent years, medical disputes have occurred one after another, and the frequent occurrence of violent injuries to doctors indicates the tension of the doctor-patient relationship. Medical disputes have become a prominent social problem, and how to improve the doctor-patient relationship has become the focus of attention from all walks of life [1]. The “Report on the Reform of China’s Medical and Health System” released by the Chinese Academy of Social Sciences shows that from 2002 to 2012, China’s medical dispute cases increased by 10 times in 10 years, and then declined, but showed an upward trend in 2016. China Hospital Association According to a new survey conducted by the Chinese Academy of Sciences, each hospital in China has an average of 27 incidents of violent injuries to doctors every year. Disputes between doctors and patients are becoming more and more serious, which has attracted the attention of governments at all levels and all sectors of society. How to properly handle medical disputes, harmonious doctor-patient relationship, and build a harmonious medical environment is not only related to the healthy development of China’s medical and health undertakings, but also to whether medicine can better serve the people’s health, and also to solve other problems during China’s transition period. The social contradictions provided some reference and reference.

In the context of the causes of doctor-patient disputes, there is a divergence in the research findings among numerous scholars. However, when viewed comprehensively, these causes primarily manifest in three aspects. Firstly, doctor-patient disputes arising from asymmetrical information are identified, with Pasca proposing, based on patient role theory, a noticeable imbalance between doctors and patients. This inequality leads to a skewed understanding of the diagnostic and treatment processes, resulting in the adversarial nature of doctor-patient relationships [2]. Some scholars also posit that information asymmetry between doctors and patients can lead to erroneous choices, ethical risks, and biased judgments, fostering the accumulation of patient dissatisfaction and transforming the doctor-patient relationship from discontent to dispute [3]. Secondly, doctor-patient disputes stemming from insufficient communication are highlighted, with Keller asserting that the underlying cause of these disputes lies in a lack of in-depth communication and dialogue [4]. Some scholars point out that medical professionals, perceiving patients as seeking medical advice, may adopt a paternalistic attitude, neglecting to value the patient’s narratives and inquiries, thereby contributing to doctor-patient disputes [5]. Thirdly, the loss of trust between doctors and patients is identified as a contributor to doctor-patient disputes. Scholars argue that as the medical profession becomes more specialized and medical institutions industrialize, trust between doctors and patients gradually erodes, ultimately leading to the occurrence of doctor-patient disputes [6]. Addressing the mitigation of doctor-patient disputes, scholars suggest that hospitals should fully leverage information systems to monitor key indicators of medical safety, establish risk sentinel warning mechanisms to address potential dispute risks [7]. Additionally, scholars propose harnessing the advantages of artificial intelligence in predicting and managing medical risks, quantifying warning indicators, and establishing risk warning systems. Hospitals should closely control potential risk factors with higher risk levels to promptly intervene in case of doctor-patient disputes [8, 9]. Hospitals should prioritize medical quality, utilizing evidence-based medicine to actively manage risks in the diagnostic and treatment processes, thereby preventing the genesis of doctor-patient conflicts at their source [10]. Governments are encouraged to play a governance role, scientifically designing the framework of laws and regulations related to doctor-patient disputes, clarifying key points such as risk prevention and patient-centric approaches [11, 12]. Furthermore, the effective role of third-party mediation mechanisms in resolving doctor-patient disputes should be fully realized [13, 14].

Most studies have qualitatively analyzed the doctor-patient relationship from various perspectives, including information asymmetry, cognitive differences between doctors and patients, trust levels, patient perceptions, and service attitudes [15, 16]. Some scholars have also examined the influencing factors of the doctor-patient relationship from the perspectives of hospitals, healthcare professionals, and government entities [17, 18]. However, there is currently a scarcity of quantitative research on the doctor-patient relationship, and few studies have adopted a quantitative approach to include hospitals, doctors, and patients in a conflict system from the perspective of decision theory. This study, based on an improved Pawlak model and grounded in systems theory, incorporates healthcare institutions, doctors, and patients as different stakeholders concerned with various disputes into the entire healthcare system. The research aims to calculate optimal solutions for collaborative development among healthcare institutions, doctors, and patients, taking into account the preferences of different stakeholders regarding disputes. This approach, exploring the causes and solutions of doctor-patient disputes from a decision theory perspective, provides a reference for alleviating doctor-patient conflicts and promoting the healthy development of the healthcare industry.

## Materials and methods

### Participants

Our study primarily investigates the conflicting interests among three stakeholders—healthcare service providers, healthcare service demanders, and healthcare service operators—in the healthcare system concerning the content, efficiency, and economic development of medical services. Given that Chinese medical institutions serve both public welfare and profit-oriented purposes, the challenge for healthcare institution managers lies in balancing the treatment effectiveness for patients, economic interests for doctors, and the generation of revenue to facilitate the overall development of the medical institution. This balancing act represents a crucial strategy for the diversified and collaborative development of medical institutions in the new century. Consequently, we extracted three decision-makers—patients, healthcare professionals, and medical institutions—in the healthcare information system. Simultaneously, we selected eight disputes and, based on the preferences of each decision-maker regarding these disputes, derived feasible solutions. These solutions serve as reference scenarios for promoting the diversified development of healthcare information systems within medical institutions.

### Data acquisition

From May to July 2022, inquiries were conducted among experts in six cities in China—Beijing, Shanghai, Yantai, Wuxi, Xiamen, and Jinan—encompassing seven tertiary hospitals and six universities. Inclusion criteria were defined as follows: ① Clinical medical experts should hold a master’s degree or higher, possess a senior professional title, and have over 10 years of work experience; ② Hospital management experts should have a bachelor’s degree or higher, engage in hospital work at tertiary hospitals for more than 5 years; ③ Medical management research experts should have a master’s degree or higher, hold an associate professorship or higher, and have over 5 years of experience in medical management research. A total of 28 experts were included in the inquiry, comprising 10 clinical medical experts, 6 hospital management experts, and 12 medical management research experts. There were 17 males and 11 females, with ages ranging from 41 to 66 years (mean age: 50 ± 4.21). Nine had master’s degrees, and 19 had doctoral degrees.

### Method introduction

**Fig. 1:**
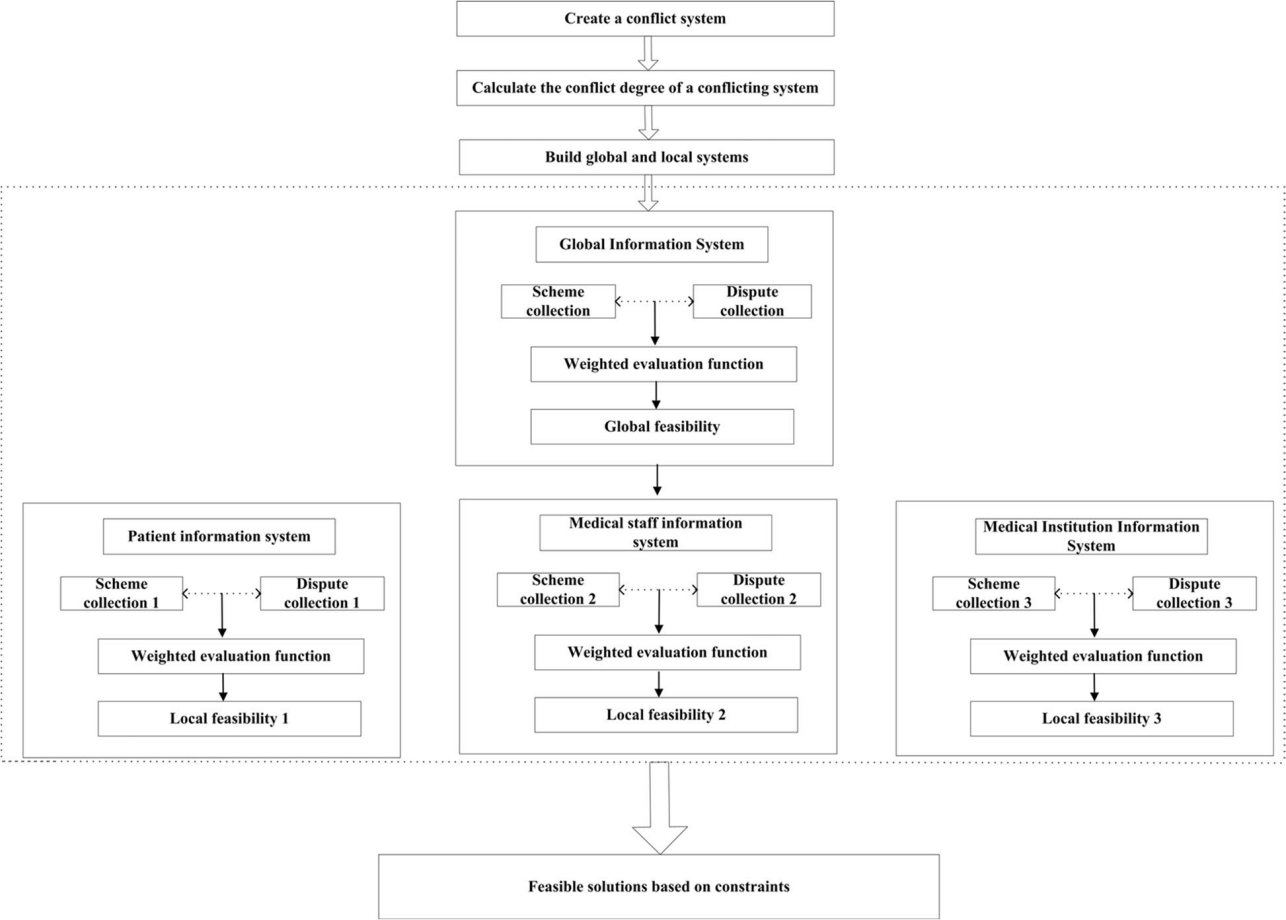
Flow chart of the multi-decision-maker mixed conflict model based on rough sets

In 1982, Z. Pawlak proposed a conflict analysis model based on rough sets, which proved to be an effective method for handling conflicts without precise information. However, this model cannot discern the specific attitudes of the conflicting parties towards a particular dispute, thus failing to understand the fundamental causes of the conflicts. Consequently, it is unable to generate satisfactory solutions agreeable to all parties involved. In pursuit of a more scientific conflict analysis approach, Professor Gao Junshan made improvements to the Pawlak conflict system model in 2008 [19]. To facilitate the diversified and collaborative development of healthcare institution information systems, it is imperative to comprehend the fundamental reasons for conflicts among hospitals, doctors, and patients, and to obtain feasible solutions satisfying all parties involved. Therefore, the modified Pawlak conflict system model was employed. Simultaneously, to overcome subjectivity in the ranking of dispute strategies inherent in the model, we incorporated the Analytic Hierarchy Process (AHP) to objectively rank dispute strategies. The model’s workflow is illustrated in Fig. 1.

#### Matrix representation of conflict intensity in the conflict system

Establish a global conflict system $S=\left(U, A\right)$, where $U=\left\{{u}_{1},{u}_{2},\cdots {u}_{n}\right\}$ is the set of decision-makers, which means that there are $n$ decision-makers ${u_1},{u_2}, \cdots {u_n}$ in the system, and $A = \{ {a_1},{a_2}, \cdots {a_k}\}$ is the set of disputes, which means that there are $k$ disputes ${a_1},{a_2}, \cdots {a_k}$ in the system. For any $a \in A$, define the attitude function of decision maker ${u_i}$ towards it as $$a({u_i}) = \left\{ \begin{array}{l} 1,{\text{ support}} \hfill \\ 0,{\text{ neutral}} \hfill \\ - 1,{\text{against}} \hfill \\ \end{array} \right.$$. This results in the voting matrix of the dispute $a$ in the system:

$${O_a} = {\left( {\begin{array}{llll} 1&{{a_{12}}}& \cdots &{{a_{1n}}} \\ {{a_{21}}}&1& \cdots &{{a_{2n}}} \\ \vdots & \vdots & \ddots & \vdots \\ {{a_{n1}}}&{{a_{n2}}}& \cdots &1 \end{array}} \right)_{n \times n}}$$

Among them, ${a_{ij}} = {a_{ji}} = \left| {a(i) + a(j)} \right| - 1$, ${a_{ij}} \in \{ 1,0, - 1\}$, and ${a_{ij}}$ take 1, -1, and 0, respectively, indicating that decision makers $i$ and $j$ have the same attitude towards dispute $a$, opposite and at least one party is neutral. Then the degree of conflict about the dispute $a$ in the system and the degree of conflict of the system are respectively:

$$\begin{aligned} Con(a)=\frac12\tau^T\left(\frac{O_{a_{ui}}\circ O_{a_{ui}}-O_{a_{ui}}}2\right)\tau,\\ Con(s) = {{~}^{{\sum\limits_{i = 1}^k {\frac{1}{2}{\tau^T}\left( {\frac{{{O_{{a_{ui}}}} \circ {O_{{a_{ui}}}} - {O_{{a_{ui}}}}}}{2}} \right)\tau } }} \! \left. \right/{\kern-0pt}\!{~}_{{\left| A \right|}}},\end{aligned}$$

Among them, $\circ$ represents the Hadamard product operation of the matrix, and $$\tau = {\left( {\begin{array}{*{20}{c}} 1&1& \cdots &1 \end{array}} \right)_{1 \times n}}^T$$ and $\left| A \right|$ represent the number of elements contained in the set $A$. The closer the $Con(s) \in \left[ {0,1} \right]$ and $Con(s)$ values are to 1, the more intense the system conflict is [19, 20].

#### Weighting of disputes in information systems and weighted evaluation function

In the global conflict system $S = (U,A)$, disputes can be classified according to certain criteria according to the characteristics of the disputes, and the assumptions can be divided into $m$ categories ${A_i} = \{ {a_1},{a_2}, \cdots ,{a_{k_i}}\} ,i = 1,2, \cdots m$, $n = \sum\limits_{i = 1}^m {k_i}$. Taking $\{ {A_i}\}$ as the first-level dispute set and $\{ {a_1},{a_2}, \cdots ,{a_{k_i}}\}$ as the corresponding second-level dispute set, the AHP method is used to calculate the weight $\{ {\omega_1},{\omega_2}, \cdots ,{\omega_n}\}$ of each dispute. In the same way, the weight $\{ {\omega ^\prime_1},{\omega ^\prime_2}, \cdots ,{\omega ^\prime_{k_i}}\}$ of each dispute in the local information system is obtained.

According to rough set theory, set the value of the decision maker ${u_j}$ for each dispute ${a_i}$ in the global conflict system as ${v_{u_j}}({a_i})$, $v({a_i}) \in \{ 0,1,2\}$, where 0, 1, and 2 indicate that the decision maker has low, medium and high requirements for the corresponding disputes, then the decision maker The weighted evaluation function (WE) of ${u_j}$ its Program ${g_l}$ is ${e_l} = \sum\limits_{i = 1}^n {v({a_i})} {\omega_i}$, In this way, the global information system $I = (U,A)$ is obtained. In the same way, the local information system ${I_j} = (U,{A_j})$, $l = 1,2, \cdots ,{3^k},j = 1,2, \cdots ,k$ of the decision maker ${u_j}$ is obtained [21–23].

#### Establishment of the conflict system

Construct the global conflict system $S = (U,A)$, determine the attitude values of decision-maker ${u_i}$ towards dispute $a_j$, and thereby establish the local system dispute set ${A_{u_i}} = \{ {a^i}|{a^i}({u_i}) \ne 0,{a^i} \in A,{u_i} \in U\}$ concerning decision-maker ${u_i}$. Calculate the conflict intensity of the conflict system using conflict intensity theory [21–23].

#### Establishment of the information system

Calculate the weights and weighted evaluation functions of each dispute in the global conflict system to obtain the global information new system $I = (U,A)$. Additionally, compute the weights and weighted evaluation functions of each dispute in the established local system dispute set to obtain various local information systems ${I_j} = (U,{A_j})$ [21–23].

#### Feasibility solution acquisition

Based on the actual circumstances of the conflict events, if additional conditions must be satisfied by the global feasible solutions, corresponding constraint conditions $h = ({a_1},{a_2}, \cdots ,{a_k})$ can be added to the global feasible solution set. The feasible solution set $\overline {\overline G } = \left\{ {g:{s_u} \in \overline {U_u} ,{s_u} \subset g,g \in G,h\left( {{f_g}({g_i},{a_1}),{f_g}({g_i},{a_2}), \cdots ,{f_g}({g_i},{a_k})} \right)} \right\}$ of the conflict system consists of solutions that simultaneously belong to both the local feasible solution set and the global feasible solution set while meeting the constraint conditions. This set represents the feasible solutions for the entire conflict event. If the feasible solution set for the conflict system is empty or contains too many solutions, thresholds $T,{T_j},j = 1,2, \cdots ,k$ can be assigned to each information system. According to the improved Pawlak theory, the feasible solution sets for each information system can be calculated, and under the constraint conditions, the feasible solutions for resolving the entire conflict event can be determined [21–23].

### Measures

#### Conflict dispute selection

The research team, considering factors such as the causes of doctor-patient disputes, the current status of their development, and strategies for mitigating such disputes, synthesized insights from the perspectives of medical quality, economic efficiency, hospital management, and development pathways [5, 24–26]. Subsequently, the team formulated four main aspects: work quality, work efficiency, economic management, and hospital development. Within these aspects, eight specific dispute contents were identified: medical quality, standardize medication, institutional efficiency, staff efficiency, hospital benefits, hospital revenue, employee development, and equipment development.

#### Expert consultation

The expert consultation questionnaire consists of three parts: ① Research purpose and questionnaire completion instructions, with a request for the return of the consultation questionnaire within 1 week; ② Preferences of each stakeholder in dispute selection. Firstly, experts are asked to assess the rationality of each indicator based on their own experience and provide modification suggestions. Secondly, experts are required to evaluate the importance of each dispute based on the preferences of each stakeholder, using a 5-point scoring system where 5 = very important, 4 = important, 3 = neutral, 2 = unimportant, 1 = very unimportant; ③ Personal information of the consulting experts, including their judgment criteria and familiarity with the evaluation indicators. Expert consultations are conducted through means such as telephone interviews, questionnaire interviews, and email feedback records.

#### Statistical analysis

Descriptive analysis was conducted using SPSS 27.0. Based on the results of expert consultations, the credibility was represented by the Expert Positivity Coefficient and the Authority Degree (Cr). The degree of agreement among expert opinions was assessed using the Kendall Concordance Coefficient (Kendall W). Additionally, Yaahp10 software was employed to calculate the weights and consistency ratios (CR) of each indicator, with a significance level set at α = 0.05.

## Results

### Descriptive statistics

Our study distributed a total of 28 questionnaires, and all 28 were effectively retrieved, resulting in a 100% response rate, indicating a high level of expert engagement. The expert judgment coefficient was 0.88, familiarity coefficient was 0.86, and authority coefficient was 0.87. With an authority coefficient of 0.7 or above, it suggests a high level of expertise among the participating experts. The Kendall’s W coefficients for the coordination of first and second-level disputes were 0.476 and 0.429, respectively (*p* < 0.001), indicating a satisfactory level of agreement among the experts.

### Voting functions of conflict parties in the medical institution conflict system

Medical institution information system $S = (U,A)$, where $U = \{ {u_1},{u_2},{u_3}\}$, ${u_1},{u_2},{u_3}$ represent patients, medical staff, medical institution, $A = \{ {a_1},{a_2},{a_3},{a_4},{a_5},{a_6},{a_7},{a_8}\}$, ${a_1},{a_2},{a_3},{a_4},{a_5},{a_6},{a_7},{a_8}$ represent medical quality, standardize medication, institutional efficiency, staff efficiency, hospital benefits, hospital revenue, employee development, equipment development. Simultaneously, the results of expert consultations were used to derive the voting functions of the three parties involved in the conflict for each dispute. The conflict system comprises three local information systems: the Patient Information System, the Medical Staff Information System, and the Medical Institution Information System. The Patient Information System involves five disputes: medical quality, standardize medication, institutional efficiency, hospital benefits, and hospital revenue. The Medical Staff Information System involves four disputes: staff efficiency, hospital benefits, hospital revenue, and employee development. The Medical Institution Information System involves six disputes: medical quality, standardize medication, institutional efficiency, hospital benefits, hospital revenue, and equipment development. The conflict intensity for the overall medical institution conflict system was calculated as 0.624 using the conflict intensity formula (see Table 1).

**Table 1:** Medical institution conflict system

	*u*_1_ patients	*u*_2_ medical staff	*u*_3_ medical institution
*a*_1_ medical quality	1	0	1
*a*_2_ standardize medication	1	0	1
*a*_3_ institutional efficiency	-1	0	1
*a*_4_ staff efficiency	0	-1	0
*a*_5_ hospital benefits	-1	1	1
*a*_6_ hospital revenue	-1	1	1
*a*_7_ employee development	0	1	0
*a*_8_ equipment development	0	0	1

### Construction of the medical institution conflict information system and weighted evaluation function results

**Fig. 2:**
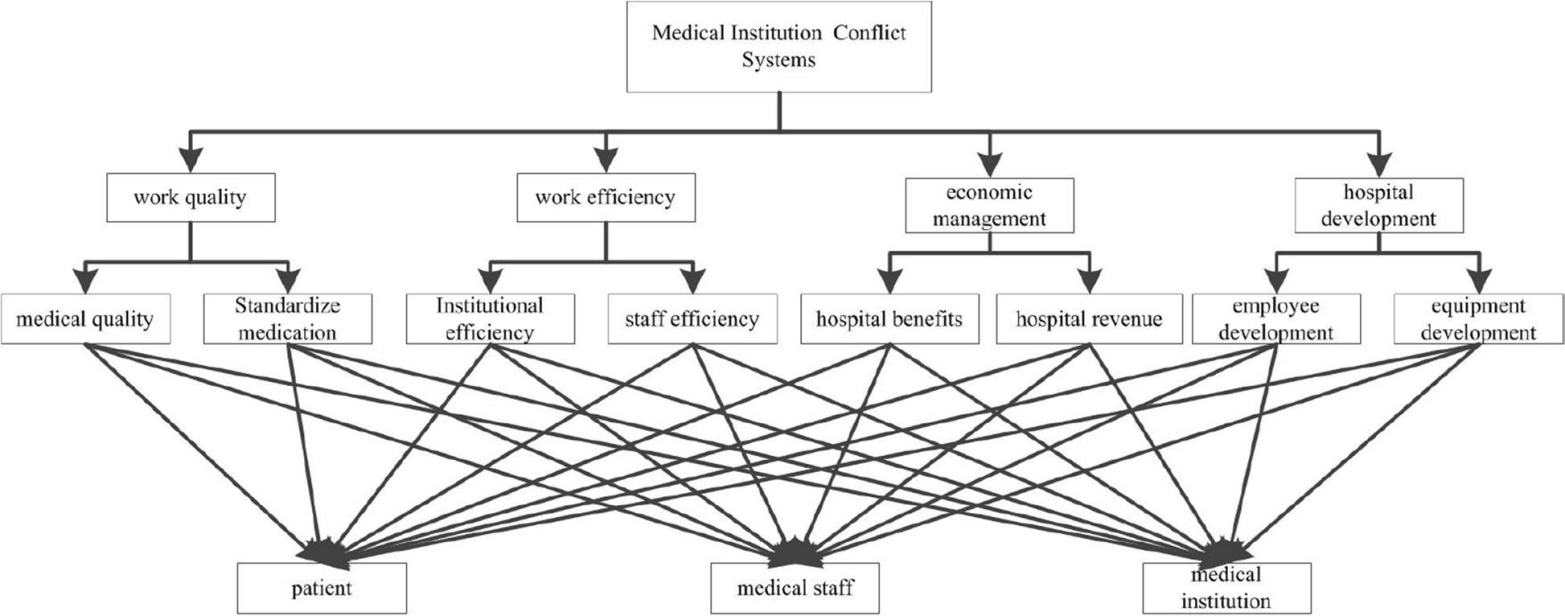
Dispute hierarchy membership model at all levels

According to the characteristics of disputes, disputes are divided into four categories: work quality, work efficiency, economic management, and hospital development. Respectively represented by ${A_1},{A_2},{A_3},{A_4}$, namely $A = \{ {A_1},{A_2},{A_3},{A_4}\}$, where are $A_1=\{a_1,a_2\},\,A_2=\{a_3,a_4\},\,A_3=\{a_5,a_6\},\,A_4=\{a_7,a_8\}$. In order to give a relatively objective evaluation function, the AHP method is used to calculate the weight of each dispute, and a hierarchical structure model is established according to the membership of the dispute set as shown in Fig. 2.

For dispute events, the Analytic Hierarchy Process (AHP) was employed based on expert ratings for each indicator. After calculating the mean values of expert ratings using the AHP method and inputting them into the Yaahp10 software, the weights of disputes within the conflict were determined. The consistency ratio (CR) values for consistency testing of judgment matrices ranged from 0 to 0.0408, all < 0.1, indicating successful consistency testing. The weights are presented in Table 2.

**Table 2:** Dispute weights

First-level disputes	Weights	Secondary disputes	Weights
Work quality	0.4918	*a*_1_ medical quality	0.3689
		*a*_2_ standardize medication	0.1230
Work efficiency	0.2459	*a*_3_ institutional efficiency	0.1844
		*a*_4_ staff efficiency	0.0615
Economic management	0.1639	*a*_5_ hospital benefits	0.0447
		*a*_6_ hospital revenue	0.0298
Hospital development	0.0984	*a*_7_ employee development	0.0656
		*a*_8_ equipment development	0.0328

In this example, due to the small number of disputes involved in each local information system, when calculating the weight of disputes in the local information system, the disputes are no longer classified and calculated by the AHP method, but the weight values in Table 2 are used. The weight of the dispute is directly applied after normalization, For example, the decision maker patient only pays attention to the three disputes of ${a_1},{a_2},{a_3}$, so the weights corresponding to ${a_1},{a_2},{a_3}$ are normalized to obtain the weight vector ${({\alpha_1},{\alpha_2},{\alpha_3})^T} =$ (0.5455,01819,0.2727)^*T*^. The weighted evaluation function of each Program in the patient’s local system can be obtained by calculating ${e_{a_g}} = \left( {{f_{a_g}}(s_{a_g}^i,{b_1}),{f_{a_g}}(s_{a_g}^i,{b_2}), \cdots ,{f_{a_g}}(s_{a_g}^i,{b_l})} \right){({\alpha_1},{\alpha_2},{\alpha_3})^T}$. The information system of decision maker ${u_1}$ (patient) is shown in Table 3. Since there are 3 types of information function values, the number of Programs increases with the number of disputes. If the number of disputes is $n$, there are ${3^n}$ Programs in total. Some programs are not in line with the actual situation or the weighted evaluation function is too low and will not become a partially feasible program. The two programs are meaningless to the information system. Therefore, only meaningful Programs are listed in the local information system table given later in this paper.

**Table 3:** Information system for decision maker ${u_1}$ (patient)

Program	Medical quality	Standardize medication	Institutional efficiency	WE
Su_1_^1^	2	2	2	2.00
Su_1_^2^	2	1	2	1.82
Su_1_^3^	2	2	1	1.73
Su_1_^4^	2	0	2	1.64
Su_1_^5^	2	1	1	1.55
Su_1_^6^	2	2	0	1.46
Su_1_^7^	1	2	2	1.45
Su_1_^8^	2	0	1	1.37
Su_1_^9^	2	1	0	1.28
Su_1_^10^	1	1	2	1.27

In the same way, the information system of decision maker ${u_2}$ (medical staff), the information system of decision maker ${u_3}$ (medical institution), and the global information system can be obtained, as shown in Tables 4, 5 and 6, respectively.

**Table 4:** Information systems for decision maker ${u_2}$ (medical staff)

Program	Hospital benefits	Hospital revenue	Employee development	WE
Su_2_^1^	2	2	2	2.00
Su_2_^2^	2	1	2	1.79
Su_2_^3^	1	2	2	1.68
Su_2_^4^	2	0	2	1.58
Su_2_^5^	2	2	1	1.53
Su_2_^6^	1	1	2	1.47
Su_2_^7^	0	2	2	1.36
Su_2_^8^	2	1	1	1.32
Su_2_^9^	1	0	2	1.26
Su_2_^10^	1	2	1	1.21
Su_2_^11^	0	1	2	1.15
Su_2_^12^	2	0	1	1.11
Su_2_^13^	2	2	0	1.06
Su_2_^14^	1	1	1	1.00

**Table 5:** Information systems for decision maker ${u_3}$ (medical institution)

Program	Medical quality	Standardize medication	Institutional efficiency	Staff efficiency	Hospital revenue	Equipment development	WE
Su_3_^1^	2	2	2	2	2	2	2.00
Su_3_^2^	2	2	2	2	1	2	1.96
Su_3_^13^	2	2	2	2	2	1	1.96
Su_3_^4^	2	2	2	1	2	2	1.92
Su_3_^5^	2	2	2	2	0	2	1.92
Su_3_^6^	2	2	2	2	1	1	1.92
Su_3_^7^	2	2	2	2	2	0	1.92
Su_3_^8^	2	2	2	1	1	2	1.88
Su_3_^9^	2	2	2	1	2	1	1.88
Su_3_^10^	2	2	2	2	0	1	1.88
Su_3_^11^	2	2	2	2	1	0	1.88
Su_3_^12^	2	1	2	2	2	2	1.85
Su_3_^13^	2	2	2	0	2	2	1.84

**Table 6:** Global information system

Program	Medical quality	Standardize medication	Institutional efficiency	Staff efficiency	Hospital benefits	Hospital revenue	Employee development	Equipment development	WE
g_1_	2	2	2	2	2	2	2	2	2.00
g_2_	1	2	2	2	2	2	2	2	1.63
g_3_	0	2	2	2	2	2	2	2	1.26
g_4_	2	1	2	2	2	2	2	2	1.88
g_5_	1	1	2	2	2	2	2	2	1.51
g_6_	0	1	2	2	2	2	2	2	1.14
g_7_	2	0	2	2	2	2	2	2	1.76
g_8_	1	0	2	2	2	2	2	2	1.39
g_9_	0	0	2	2	2	2	2	2	1.02
g_10_	2	2	1	2	2	2	2	2	1.82
g_11_	1	2	1	2	2	2	2	2	1.45
g_12_	0	2	1	2	2	2	2	2	1.08
g_13_	2	1	1	2	2	2	2	2	1.7
g_14_	1	1	1	2	2	2	2	2	1.33
g_15_	0	1	1	2	2	2	2	2	0.96
g_16_	2	0	1	2	2	2	2	2	1.57
g_17_	1	0	1	2	2	2	2	2	1.20
g_18_	0	0	1	2	2	2	2	2	0.83
g_19_	2	2	0	2	2	2	2	2	1.63
g_20_	1	2	0	2	2	2	2	2	1.27
g_21_	0	2	0	2	2	2	2	2	0.90
g_22_	2	1	0	2	2	2	2	2	1.51
g_23_	1	1	0	2	2	2	2	2	1.14
g_24_	0	1	0	2	2	2	2	2	0.77
g_25_	2	0	0	2	2	2	2	2	1.39
g_26_	1	0	0	2	2	2	2	2	1.02

### Feasibility solution results of the medical institution conflict information system

The constraints of the medical institution conflict system include $$\left\{ \begin{array}{l} {a_4} + {a_7} \leq 3 \hfill \\ {a_5} + {a_8} \leq 3 \hfill \\ \end{array} \right.$$. According to the results of the expert correspondence, given ${T_{u_1}} = 1.6$,${T_{u_2}} = 1.55$ and ${T_{u_3}} = 1.9$. Therefore, the ultimate feasible solution for the medical institution conflict system is determined to be:

$$\overline {\overline G } = \{ (2,2,2,2,2,2,1,1),(2,2,2,1,1,2,2,2)\}$$

In other words, the feasible solution sequence for the medical institution conflict system, ranked from high to low, is as follows: high medical quality, high standardize medication, high institutional efficiency, high staff efficiency, high hospital benefits, high hospital revenue, medium employee development, and medium equipment development. Alternatively, the optimal state where all three parties involved in the medical institution system are satisfied is characterized by high medical quality, high standardize medication, high institutional efficiency, medium staff efficiency, medium hospital benefits, high hospital revenue, high employee development, and high equipment development.

## Discussions

This paper extends the bilateral conflict system of the modified Pawlak conflict system model to a multilateral conflict system. The subjective evaluation function in the conflict system is replaced with a weighted evaluation function, enhancing the systematic and objective nature of conflict resolution results. Ultimately, by considering constraint conditions and the logical relationships among various local feasible solution sets, the optimal strategy for the collaborative development of each stakeholder in the medical institution system is determined.

The research results reveal that both indicators in “work quality” need to be highly valued. Overall, emphasizing “medical quality” and “standardize medication” contributes to establishing a safe, efficient, and reliable healthcare system, which significantly influences the sustainable development of the healthcare system. For patients, “medical quality” and “standardize medication” directly relate to their life safety and health. For medical institutions, “standardize medication” and high-quality medical care help avoid unnecessary medical expenses and resource wastage. Optimizing the allocation of medical resources and improving the efficiency of medical services can be achieved through the rational use of drugs, tests, and treatment methods. For doctors, enhancing “medical quality” and “standardize medication” can build trust in patients towards the healthcare system. Patients are more willing to accept medical services, adhere to medical advice, thereby promoting a positive development in doctor-patient relationships. In the first scenario, “employee development” and “equipment development” need to be at a moderate level. From the perspective of patients, focusing on the development of medical staff and hardware equipment inevitably reduces the humanistic care for patients. Meanwhile, charging higher medical fees supports the transformation and upgrading of medical equipment. From the perspective of doctors, when the hospital achieves high efficiency and income, and their income level is guaranteed, “employee development” will not be a top priority. From the viewpoint of the medical institution, having high “medical quality”, “institutional efficiency”, and “hospital revenue” is crucial for creating the brand effect of the medical institution and maintaining trust between doctors and patients, with “hospital development” placed as a secondary concern.In the second scenario, “staff efficiency” and “hospital benefits” are at a moderate level. Firstly, patients expect high-quality outcomes. When “medical quality” and “standardize medication” reach high levels, moderate “staff efficiency” is acceptable to patients. When “hospital benefits” are at a moderate level and high “medical quality” is required, doctors prefer high “employee development” and hope to reduce “staff efficiency”. After having standardized medical technology, work efficiency, and sufficient “hospital development”, medical institutions can accept some loss of benefits to achieve the optimal strategy for the collaborative development of doctors, patients, and management.

### Higher medical quality and highly standardized medication are the core of promoting doctor-patient relationship

Medical quality indicators are the focus of all stakeholders, and medical institutions must maintain high medical quality and standardize medication. With the implementation of the Healthy China Strategy, people’s health and safety has become the focus of attention. As a carrier of people’s health, the improvement of the internal management level of medical institutions is not only a major agenda to promote the development of medical and health care, but also to improve the people’s health. Necessary measures for healthy lifespan [27] Therefore, it is necessary to further implement the refined management of medical institutions, strengthen the supervision of medical quality, start from the whole process of diagnosis and treatment, medication, and rehabilitation, effectively control medical accidents, and take emergency prevention and control measures [28]. In addition, the policy of “separation of medicines” is strictly implemented to provide patients with high-quality medical services and improve the quality of life of patients.

### Pay attention to the economic management of the hospital to improve the professional identity of medical staff

Medical institutions should pay attention to the economic management of hospitals on the premise of ensuring high quality and efficient work. The economic effect of a hospital can not only create better hardware facilities for medical institutions, but also improve the treatment level of medical staff, thereby strengthening their professional identity [29]. The economic benefits of medical institutions are mainly from two aspects: cost control and word-of-mouth benefits. Cost control is mainly reflected in three aspects: operating costs, daily expenses and risk expenses [30]. Operating cost control can start from purchasing cost control, and can implement the separation of five rights of supplier selection, pricing, quantitative, payment and acceptance, and strictly control the cost of drugs and consumables. At the same time, it can improve the technical level of medical staff, pay attention to the development of medical staff, and provide them with training opportunities in a timely manner, thereby effectively improving the efficiency of their medical services and reducing risk costs. Word-of-mouth benefits can enhance the image of the hospital through online and offline channels, actively communicate with patients, and enhance patients’ trust.

### Strengthening the “soft and hard” environment construction of medical institutions is the foundation of coordinated development

Our research also found that hospital development should be placed in a secondary position, taking into account medical employee development and efficiency, and at the same time attaching importance to the construction of the “soft and hard” environment of medical institutions, and medical staff should strengthen humanistic care to create a good doctor-patient service. At the same time, the medical institution can popularize medical knowledge through the official website and WeChat platform, build a positive transmission channel for medical information, and promote the relative symmetry of doctor-patient information [31]. In addition, it is necessary to pay attention to the upgrading of hospital equipment, reduce the patient’s consultation process, shorten the patient’s inspection time, and improve the comfort of inspection and inspection, thereby reducing the probability of doctor-patient conflict.

### Scientific and practical aspects of the medical institution conflict system

The reliability of the study depends on the research methodology and outcomes. This paper first constructs a multi-decision-maker hybrid conflict model for the medical institution information system. To achieve a more objective and scientific evaluation function, the matrix form of the conflict theory in the improved Pawlak model is employed. Secondly, from the perspective of systems theory, incorporating medical institutions, healthcare professionals, and patients as different stakeholders concerned with various disputes into the entire healthcare system, we establish a medical institution conflict system involving three stakeholders. The hybrid conflict model is applied to the conflict system involving doctors, patients, and management to determine the optimal solution for resolving healthcare conflicts. The goal is to find a balance point in the relationships among various entities in the medical institution information system, thereby minimizing conflicts, mitigating doctor-patient tensions, and seeking the optimal solution to address healthcare conflicts. Additionally, from the perspective of stakeholder theory, this paper establishes a multi-decision-maker hybrid conflict model for the medical system. By deriving feasible solutions satisfying all three conflicting parties, it proposes an approach for the collaborative governance of the healthcare service system, providing decision-making references to promote the sustainable development of the healthcare industry.

## Limitations

Our research also has certain limitations. Firstly, there are significant differences in healthcare conflicts among various medical institutions in different regions. Due to limitations imposed by objective conditions, this study did not conduct in-depth research on the definition and values of information variables. Secondly, given the complexity of the factors involved, the approach to resolving conflicts in healthcare institutions only established a theoretical analytical model. In terms of indicator selection, only four aspects with a total of eight indicators were included, potentially overlooking key factors. It is worth noting that this study also has the limitation of lack of discussion and application of real-world data cases. In future research, more consideration needs to be given to scientific index construction, clear relationships between stakeholders, and the use of typical case provides an in-depth analysis of the scientific nature and practicality of the model.

## Conclusions

In our paper, the two-party conflict system in the improved Pawlak conflict system model is extended to a multi-party conflict system, and the subjective evaluation function in the conflict system is changed to a weighted evaluation function, the systemicity and objectivity of conflict resolution results are enhanced. Finally, the optimal strategy for the coordinated development of various stakeholders in the medical institution system is obtained through the constraints and the logical relationship between the local feasible Program sets. The following conclusions are drawn: (1) The conflict intensity of the overall healthcare institution conflict system is 0.624, indicating that conflicts in the healthcare institution system still require ongoing attention. (2) The medical quality index is the focus of all stakeholders, and medical institutions should maintain high medical quality and standardize medication; (3) Medical institutions should ensure high quality and efficient work. Pay attention to the economic management of the hospital; (4) Put hospital development in a secondary position, and pay attention to the renewal of medical equipment while taking into account medical employee development and efficiency.

## Data Availability

The data sets used and/or analyzed during the current study are available from the corresponding author on reasonable request.
